# Low Socioeconomic Status Is Associated with Worse Outcomes After Curative Surgery for Colorectal Cancer: Results from a Large, Multicenter Study

**DOI:** 10.1007/s11605-019-04435-2

**Published:** 2019-11-19

**Authors:** I. van den Berg, S. Buettner, R. R. J. Coebergh van den Braak, K. H. J. Ultee, H. F. Lingsma, J. L. A. van Vugt, J. N. M. Ijzermans

**Affiliations:** 1 Department of Surgery, Erasmus MC - University Medical Center, Rotterdam, The Netherlands; 2 Department of Public Health, Erasmus MC - University Medical Center, Rotterdam, The Netherlands

**Keywords:** Colorectal cancer, Surgical resection, Socioeconomic status, Postoperative outcomes, Survival

## Abstract

**Background:**

Socioeconomic status (SES) has been associated with early mortality in cancer patients. However, the association between SES and outcome in colorectal cancer patients is largely unknown. The aim of this study was to investigate whether SES is associated with short- and long-term outcome in patients undergoing curative surgery for colorectal cancer.

**Methods:**

Patients who underwent curative surgery in the region of Rotterdam for stage I–III colorectal cancer between January 2007 and July 2014 were included. Gross household income and survival status were obtained from a national registry provided by Statistics Netherlands Centraal Bureau voor de Statistiek. Patients were assigned percentiles according to the national income distribution. Logistic regression and Cox proportional hazard regression were performed to assess the association of SES with 30-day postoperative complications, overall survival and cancer-specific survival, adjusted for known prognosticators.

**Results:**

For 965 of the 975 eligible patients (99%), gross household income could be retrieved. Patients with a lower SES more often had diabetes, more often underwent an open surgical procedure, and had more comorbidities. In addition, patients with a lower SES were less likely to receive (neo) adjuvant treatment. Lower SES was independently associated with an increased risk of postoperative complications (Odds ratio per percent increase 0.99, 95%CI 0.99–0.998, *p* = 0.004) and lower cancer-specific mortality (Hazard ratio per percent increase 0.99, 95%CI 0.98–0.99, *p* = 0.009).

**Conclusion:**

This study shows that lower SES is associated with increased risk of postoperative complications, and poor cancer-specific survival in patients undergoing surgery for stage I–III colorectal cancer after correcting for known prognosticators.

## Introduction

With an incidence of 464,800 patients in 2012, colorectal cancer (CRC) is the second most common cancer in Europe and constitutes a large burden, both in economic and in medical terms.[1, 2] In a recent European article, average costs for treating a single patient with colorectal cancer were estimated at 40,000 Euros [2]. Currently, the TNM classification is the most important determinant for treatment decisions and outcome. Resection still remains the only cure, and the 5-year survival rate for patients with stage I–III varies between 53 and 92% [1]. Still, there are individual differences in clinical outcome within a single tumour stage [3]. Next to these tumour characteristics, several patient factors such as obesity, diabetes mellitus, smoking and nutritional status have been investigated and associated with survival, yet much of the disparity in prognosis remains unexplained [4–7].

A possible explanation for differences in survival is socioeconomic status (SES). Not only does this influence the ability to pay medical bills and having financial resources to follow through with all hospital check-ups but also other correlating factors such as obesity and diabetes may also have an influence on post-resection CRC survival [8–10]. In previous studies, SES has been investigated as a possible prognostic factor for survival in cancer patients with contradicting results [11–23]. Some of this variability in outcomes may be explained by accessibility of healthcare [22, 23]. Some studies have been performed in countries where access to and the quality of healthcare is directly linked to income (e.g. USA) [22, 23], whereas others were conducted in countries with equal healthcare access (e.g. Great Britain and Scandinavia) [11–23]. The Dutch healthcare system is known for its equal healthcare access,[24, 25] meaning that differences in outcome associated with SES can be attributed to patient and provider factors and their interaction, rather than disparities in healthcare [26]. In the current study, we aimed to explore the association between SES, as assessed by household income, and outcomes following curative resection for stage I–III colorectal cancer in the Netherlands.

## Methods

### Study Population

Patients with stage I–III colorectal cancer who underwent curative surgery, enrolled in the MATCH-study between 2007 and 2014, were included in this study. The MATCH study is a prospective multicentre cohort study including patients to obtain fresh frozen CRC tissue samples with matched clinical data from 2007 until December 2017 in six hospitals in the region of Rotterdam [27].

The rationale of the MATCH study was to identify subtypes of colorectal cancer, related prognostic markers and outcome of treatment [28]. The study was approved by the Erasmus MC IRB (MEC-2007-088) and all patients provided written informed consent.

### Study Parameters

Socioeconomic status was defined as gross household income (GHI), the most commonly used and accepted surrogate marker for SES [26, 29–31]. GHI from the year prior to surgery was used for analyses, as the income in the year of surgery was possibly lower due to disease-related absence from work. Annual earnings were obtained from Statistics Netherlands, a governmental organisation enabling studies on social issues on the basis of reliable statistical information (Centraal Bureau voor de Statistiek; CBS), including all types of income of people sharing a household or place of residence combined (i.e. salary, state pension, social compensation, and investment revenues). Patients were assigned percentiles and quartiles (Q1–Q4) according to the national income distribution (i.e. patients of a household with an annual salary corresponding to 0–25% of the GHI of the Dutch population were stratified to the first income quartile). Baseline characteristics and variables were retrieved from the prospective database of the MATCH study.

### Dutch Healthcare System

The current Dutch healthcare system was introduced on January 1, 2006. All Dutch citizens are legally required to have healthcare insurance offered by several private insurers. Basic insurance premiums have a legal maximum and allowances are available for the lower incomes. Basic insurances cover all medical costs concerning regular cancer care including all in hospital care, costs for medicines, cancer rehabilitation, emergency transfer to hospitals, dietary help and psychosocial assistance. Out-of-pocket expenses such as transportation represented 14.7 percent of healthcare spending in 2014 [32].

### Outcome Measures

The primary endpoints were overall survival (OS) and cancer-specific survival (CSS) after surgery for colorectal cancer, calculated from the day of surgery to the day of death (from disease) or loss to follow-up, whichever came first. Date and cause of death were obtained from the national registry of Statistics Netherlands. Secondary outcome measures were 30-day postoperative complications. Severity of complications was scored according to the Clavien-Dindo classification, and major 30-day postoperative complications were defined as Clavien-Dindo score ≥ 3a [33].

### Statistical Methods

Descriptive statistics and multivariable analyses were performed using SPSS version 24.0 (SPSS, Inc., Chicago, IL, USA). Differences in baseline characteristics between the SES quartiles were tested with Pearson’s chi-square analysis or Mann-Whitney U-test as appropriate. Logistic regression analysis was used to calculate the odds ratio (OR) with 95% confidence intervals (95% CI) to evaluate the influence of SES, patient and tumour characteristics and operation techniques on 30-day postoperative complications. The outcomes OS and CSS were analysed with Cox proportional hazards regression. Unadjusted differences in the survival between income quartiles were assessed using the log-rank test. The predictors included in regression models were selected based on clinical relevance based on previous literature. Two-sided *p* values < 0.05 were considered statistically significant.

## Results

A total of 975 patients met the inclusion criteria. For 10 patients, gross household income (GHI) could not be retrieved, leaving a final sample size of 965 (99%) patients. Patients with low SES were more often female and older (both *p* < 0.001) (Table 1). Diabetes mellitus and higher American Society of Anesthesiologists (ASA) classification were more common among patients with low SES (both *p* = 0.001). The Charlson comorbidity index was significantly higher for patients in the lowest quartile (*p* < 0.001). The median length of stay (LOS) was higher for patients in the lowest quartile (*p* < 0.001). Treatment strategies also differed between the quartiles; patients with low SES more often underwent open surgery (*p* = 0.003) and less often received neoadjuvant and adjuvant treatment (*p* = 0.037 and *p* = 0.001, respectively).

**Table 1:** Baseline characteristics

	Q1	%	Q2	%	Q3	%	Q4	%	*p* value
	(*n* = 244)		(*n* = 348)		(*n* = 187)		(*n* = 186)		
Demographics									
Sex female	146	(59.8)	153	(44)	75	(40.1)	63	(33.9)	< 0.001
Age (median, IQR)	76	(69–81)	72	(66–78)	66	(60–72)	59	(55–65)	< 0.001
BMI (median, IQR)	25.9	(23.3–29.4)	25.9	(23.5–28.5)	25.8	(23.0–28.3)	25.4	(23.4–28.7)	0.795
CEA preoperative (median, IQR)	3.3	(2.0–8.0)	3.5	(2.0–8.0)	3.3	(1.85–6.9)	2.9	(1.9–5.75)	0.160
Comorbid conditions									
Charlson comorbidity index (median, IQR)	1	(0.0–2.0)	1	(0–2.0)	0	(0–1.0)	0	(0–0.1)	< 0.001
Diabetes mellitus	62	(25.5)	72	(20.7)	28	(15)	20	(10.8)	0.001
COPD	27	(11.1)	29	(8.3)	14	(7.5)	12	(6.5)	0.344
ASA									
• I–II	189	(78.1)	274	(79)	159	(86.4)	168	(90.8)	0.001
• III+	53	(21.9)	73	(21)	25	(13.6)	17	(9.2)	
• Missing	2		1		3		1		
Surgical technique									
• Open	121	(49.8)	154	(44.3)	66	(35.9)	77	(41.6)	0.003
• Laparoscopic	102	(42)	168	(48.3)	105	(57.1)	80	(43.2)	
• Conversion	20	(8.2)	26	(7.5)	13	(7.1)	28	(15.1)	
• Missing	1		0		3		1		
Stoma	69	(38.3)	106	(30.5)	61	(33)	55	(29.7)	0.768
• Missing	0		1		2		1		
Length of stay (LOS)	9	(6–14)	7.5	(6–13)	7	(5–10)	7	(5–12)	< 0.001
Blood loss (L)	0.159	(0.05–0.40)	1	(0.03–0.40)	0.11	(0.04–0.25)	0.1	(0.02–0.35)	0.065
Operation duration (hours)	2.53	(1.83–3.28)	2.47	(1.83–3.25)	2.5	(2.0–3.2)	2.67	(1.87–3.52)	0.356
Neoadjuvant therapy	57	(23.4)	84	(24.2)	63	(33.7)	57	(30.6)	0.037
Adjuvant chemotherapy	28	(11.6)	60	(17.3)	40	(21.4)	50	(26.9)	0.001
• Missing	3		2		0		0		
Tumour characteristics									
Tumour stage									
• I	82	(33.6)	107	(30.7)	52	(27.8)	64	(34.4)	0.278
• II	82	(33.6)	139	(39.9)	69	(36.9)	56	(30.1)	
• III	80	(32.8)	102	(29.3)	66	(35.3)	66	(35.5)	
Tumour grade differentiation									
• Good	59	(25.5)	89	(26.6)	54	(29.7)	48	(26.4)	0.534
• Moderate	144	(62.3)	214	(64.1)	105	(57.7)	120	(65.9)	
• Poor	28	(12.1)	31	(9.3)	23	(12.6)	14	(7.7)	
• Missing	13		14		5		4		
Angioinvasion									
• No	65	(28.8)	94	(28.8)	60	33.9)	57	(32.8)	0.749
• Yes	25	(11.1)	29	(8.9)	15	8.5)	13	(7.5)	
• Not reported	136	(60.2)	203	(62.3)	102	57.6)	104	(59.8)	
• Missing	18		22		10		12		
Location of tumour rectum	64	(26.2)	99	(28.4)	66	(35.3)	67	(36)	0.059

### SES and Postoperative Complications

**Figure 1:**
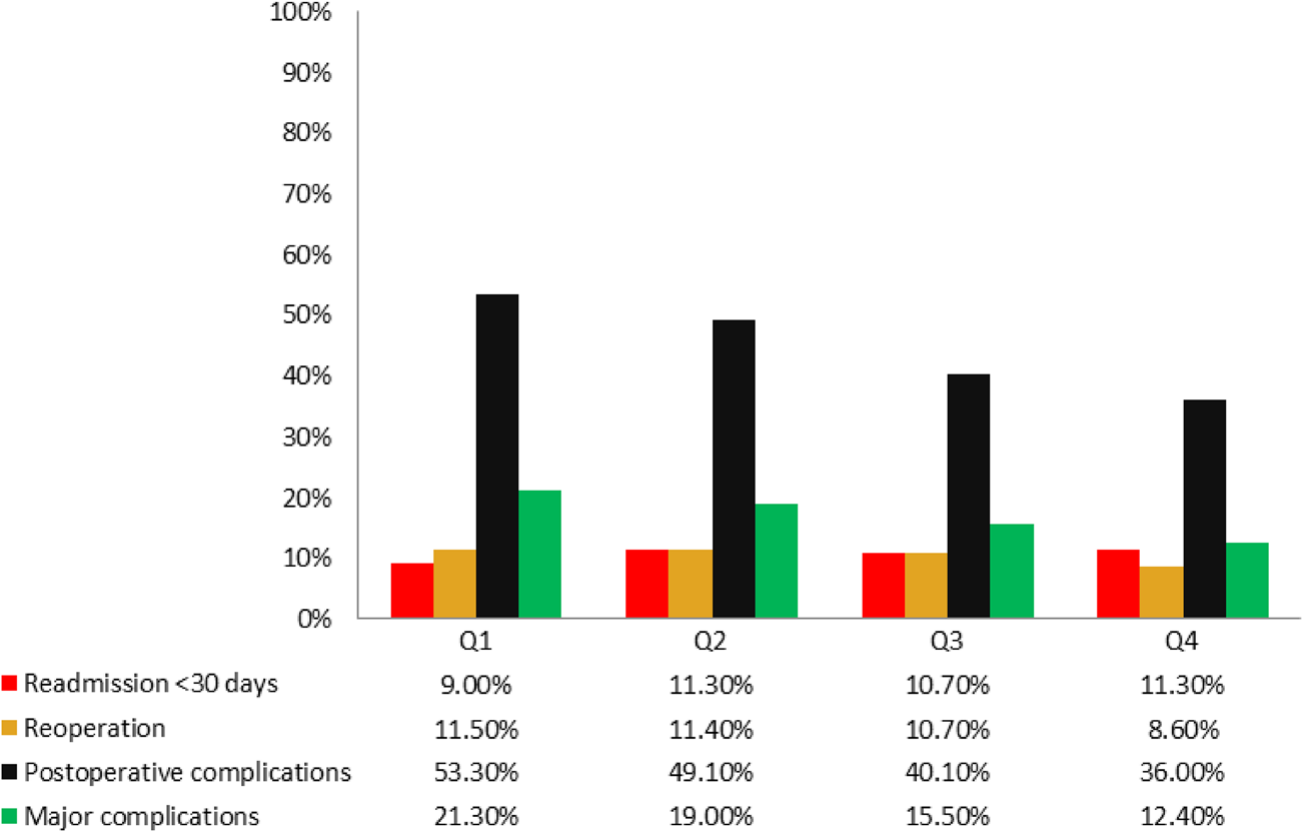
Postoperative complications per quartile

A total of 443 patients (45.9%) suffered at least one postoperative complication. The overall complication rate gradually decreased from 53.3% in Q1 to 36.0% in Q4 (*p* < 0.001). Major complications (i.e. Clavien-Dindo score ≥ 3) occurred in 170 patients (17.6%), for which a similar gradual decrease was observed (21.3% in Q1 to 12.4% in Q4; p < 0.001). No significant differences in readmission and reoperation rates were observed between the quartiles (Fig. 1).

In univariable logistic regression, GHI was associated with overall postoperative complications (OR 0.99, 95%CI 0.99 – 0.99, *p* < 0.001), as was older age (OR 1.02, 95%CI 1.01–1.04, *p* < 0.001), rectal cancer (OR 1.56, 95%CI 1.19–2.05, *p* = 0.001), and open surgery (OR 0.56, 95% CI 0.44–0.73, *p* < 0.001). The association between GHI and overall postoperative complications remained significant in the multivariable model (OR 0.99, 95%CI 0.99–0.998, *p* = 0.004) (Table 2). For major postoperative complications, no independent association was found in multivariable analysis (OR 0.99, 95% CI 0.99–1.00, *p =* 0.103) (Table 3).

**Table 2:** Univariable and multivariable analyses on overall postoperative complications

	Odds ratio	Univariable			Multivariable	
		95% confidence interval	*p* value	Odds ratio	95% confidence interval	*p* value
Gender–male = reference	0.84	0.65–1.08	0.174	0.79	0.60–1.00	0.086
Age (years)	1.02	1.01–1.04	< 0.001	1.02	1.00–1.03	0.044
Charlson comorbidity	1.09	1.00–1.19	0.051	1.03	0.94–1.00	0.497
Rectal carcinoma	1.56	1.19–2.05	0.001	1.81	1.00–2.00	< 0.001
Surgical technique Open = reference	0.56	0.44–0.73	< 0.001	0.56	0.42–0.73	< 0.001
Gross household income	0.99	0.99–0.99	< 0.001	0.99	0.99–0.998	0.004

**Table 3:** Univariable and multivariable analyses on major postoperative complications (Clavien Dindo ≥ 3 )

		Univariable			Multivariable	
	Odds ratio	95% confidence interval	*p* value	Odds ratio	95% confidence interval	*p* value
Gender–male = reference	0.83	0.60–1.16	0.283	0.85	0.6–1.20	0.354
Age (years)	1.02	1.01–1.04	0.009	1.01	1.00–1.03	0.142
Charlson comorbidity	1.13	1.02–1.25	0.017	1.08	0.97–1.20	0.187
Rectal carcinoma	1.54	1.09–2.17	0.014	1.72	1.19–2.47	0.004
Surgical technique–open procedure = reference	0.47	0.34–0.66	< 0.001	0.47	0.33–0.67	< 0.001
Gross household income	0.99	0.99–0.998	0.009	0.99	0.99–1.00	0.103

### SES and Overall Survival

**Figure 2:**
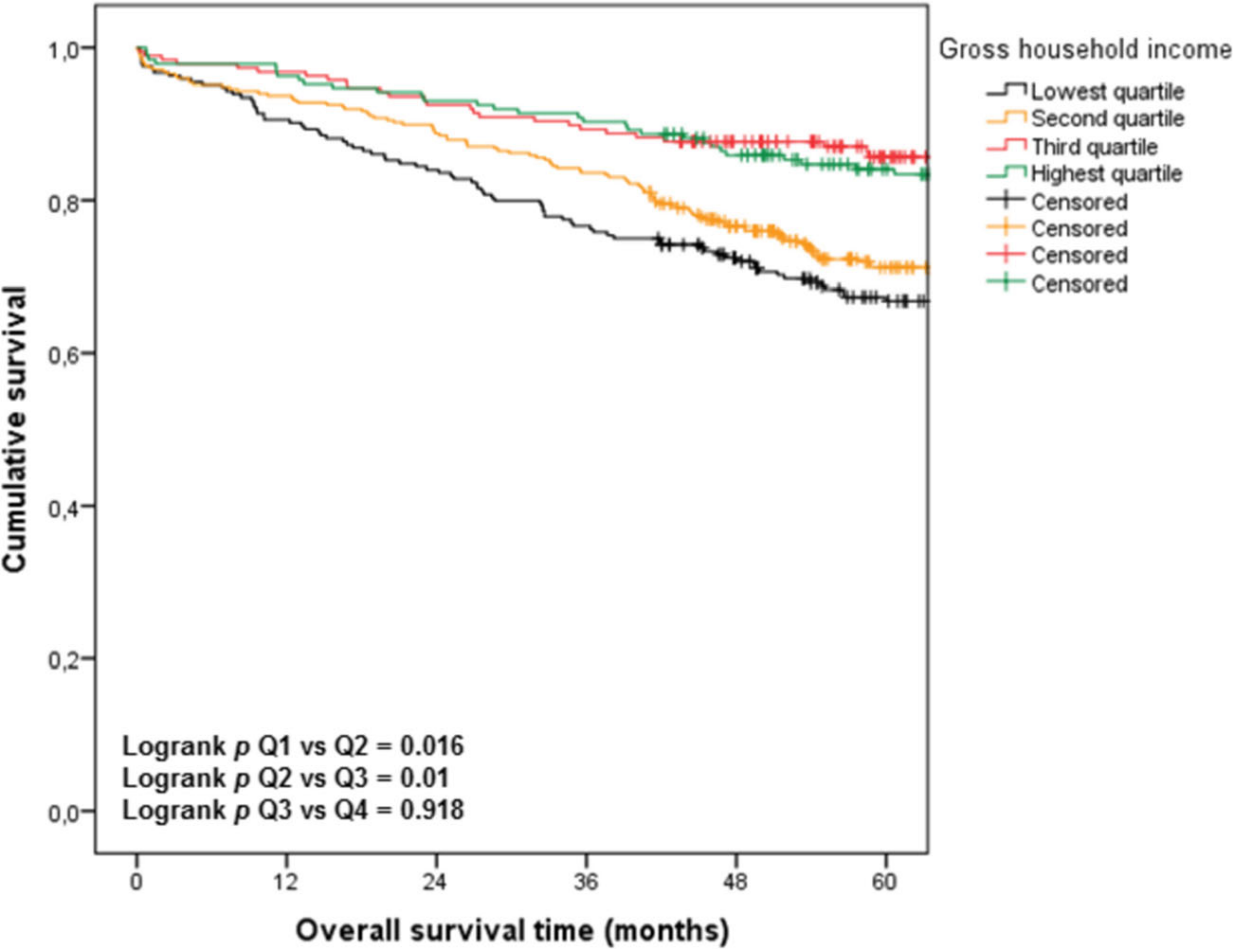
Overall 5 year survival per socioeconomic income quartile

OS increased gradually with increasing socioeconomic status (Fig. 2**)**. The median OS was only reached in Q1 (88.9 months, 95% CI 79.1–98.7), whereas the median OS was not reached in any of the other quartiles. Patients in Q1 had a worse OS compared with patients in Q2 (*p* = 0.016) and patients in Q2 had a worse survival compared with patients in Q3 (*p* = 0.01). Patients in Q3 and Q4 had a similar survival (*p* = 0.918).

In univariable analysis, GHI was associated with OS (HR 0.98, 95% CI 0.98–0.99, *p* < 0.001). Furthermore, age (HR 1.04, 95% CI 1.02–1.06, *p* < 0.001), CCI (HR 1.24, 95% CI 1.17–1.32, *p* < 0.001), tumour stage (HR 1.90, 95%CI 1.42–2.55, *p* < 0.001) and tumour grade (HR 1.89, 95%CI 1.29–2.76, *p* = 0.001) were associated with OS. In the multivariable analysis, GHI was not independently associated with OS (HR 0.99, 95%CI 0.99-1.00, *p* = 0.158), in contrast to age (HR 1.07, 95%CI 1.05–1.08, *p* < 0.001), CCI (HR 1.17, 95%CI 1.10–1.25, *p* < 0.001) tumour stage (HR 2.34, 95%CI 1.72–3.19, *p* < 0.001) and tumour grade (HR 1.53, 95% CI 1.04–2.26, *p* = 0.030) (Table 4).

**Table 4:** Univariable and multivariable Cox regression analyses on overall survival

	Univariable			Multivariable		
	Hazard ratio	95% confidence interval	*p* value	Hazard ratio	95% confidence interval	*p* value
Age	1.04	1.02–1.06	< 0.001	1.07	1.05–1.08	< 0.001
Charlson comorbidity index	1.24	1.17–1.32	< 0.001	1.17	1.10–1.25	< 0.001
Tumour stage I (Ref)	-	-	-	-	-	-
Tumour stage II	1.26	0.93–1.71	0.138	1.20	0.88–1.65	0.253
Tumour stage III	1.90	1.42–2.55	< 0.001	2.34	1.72–3.19	< 0.001
Tumour grade good (Ref)	-	-	-	-	-	-
Tumour grade moderate	0.87	0.65–1.16	0.340	0.81	0.60–1.09	0.167
Tumour grade poor	1.89	1.29–2.76	0.001	1.53	1.04–2.26	0.030
Gross household income	0.98	0.98–0.99	< 0.001	0.99	0.99–1.00	0.158

### SES and Cancer-Specific Survival

**Figure 3:**
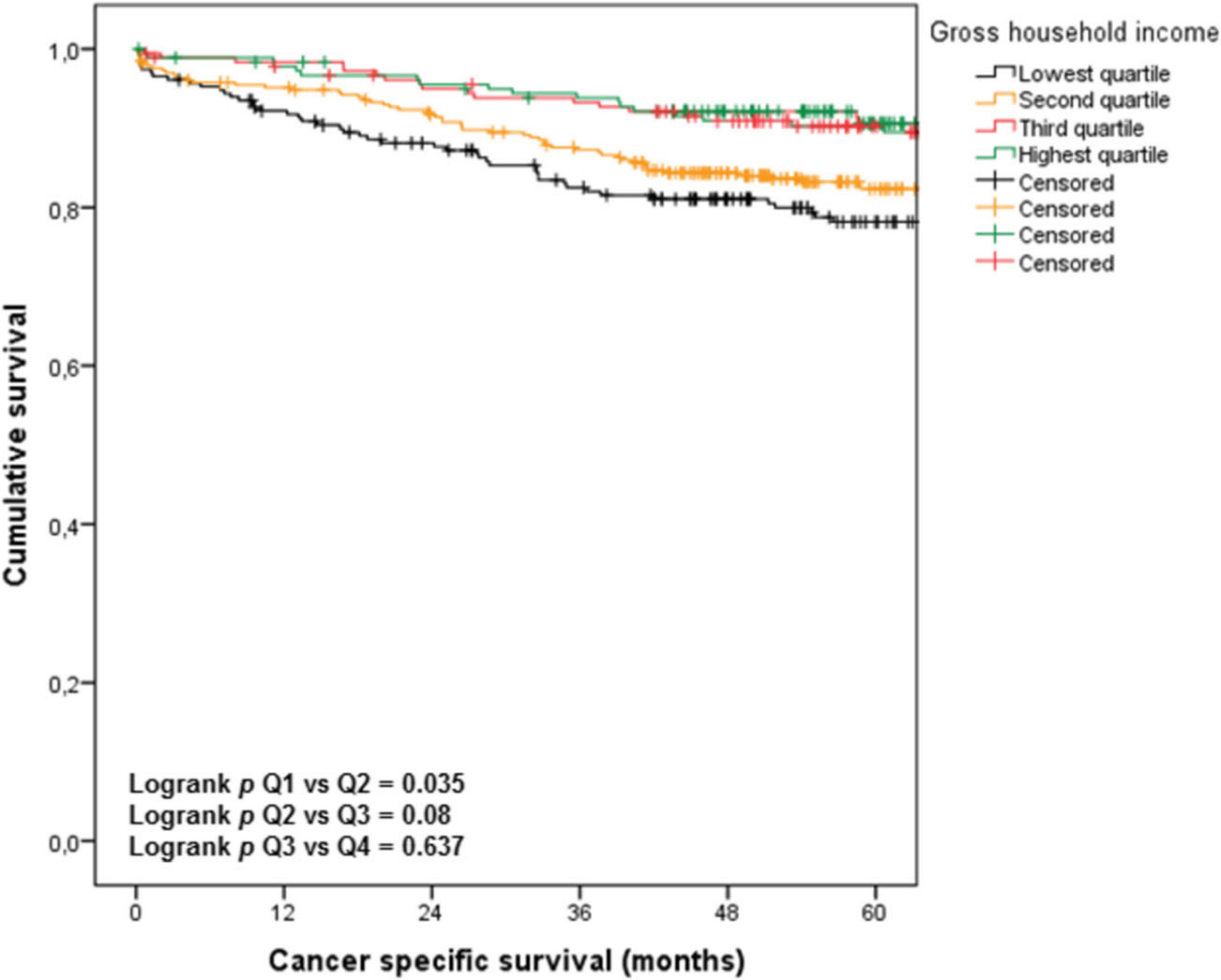
Cancer specific survival per socioeconomic income quartile

The median CSS was not reached for any of the quartiles. Patients in Q1 had a worse CSS compared with patients in Q2 (*p* = 0.035). No difference in survival was found between Q2 and Q3, nor for Q3 and Q4 (*p* = 0.080 and *p* = 0.637, respectively) (Fig. 3).

In the univariable model for CSS, GHI showed a significant association (HR 0.98, 95%CI 0.98–0.99, *p* < 0.001) as well as age (HR 1.04, 95%CI 1.02–1.06, *p* < 0.001), CCI (HR 1.14, 95%CI 1.04–1.25, *p* = 0.006), tumour stage (HR 3.58, 95% CI 2.31–5.56, *p* < 0.001) and tumour grade (HR 2.62, 95% CI 1.52–4.52, *p* = 0.001). The association between GHI and CSS remained statistically significant in the multivariable model (HR 0.99, 95%CI 0.98–0.99, *p* = 0.009). Other factors that were independently associated with CSS were age (HR 1.03, 95%CI 1.01–1.05, *p* = 0.001) and tumour stage (HR 3.95, 95%CI 2.49–6.28, *p* < 0.001) (Table 5).

**Table 5:** Univariable and multivariable Cox regression analysis on cancer specific survival

	Univariable			Multivariable		
	Hazard ratio	95% confidence interval	*p* value	Hazard ratio	95% confidence interval	*p* value
Age	1.04	1.02–1.06	< 0.001	1.03	1.01–1.05	0.001
Charlson comorbidity index	1.14	1.04–1.25	0.006	1.07	0.96–1.18	0.214
Tumour stage I (Ref)	-	-	-	-	-	-
Tumour stage II	1.33	0.81–2.19	0.262	1.28	0.76–2.16	0.353
Tumour stage III	3.58	2.31–5.56	< 0.001	3.95	2.49–6.28	< 0.001
Tumour grade good (Ref)	-	-	-	-	-	-
Tumour grade moderate	1.19	0.77–1.84	0.430	1.09	0.71–1.69	0.694
Tumour grade pore	2.62	1.52–4.52	0.001	1.84	1.06–3.19	0.031
Gross household income	0.98	0.98–0.99	< 0.001	0.99	0.98–0.997	0.009

## Discussion

The results of this study show that lower SES is significantly associated with worse outcome in patients with stage I–III colorectal cancer undergoing curative surgery. We observed an increased rate of postoperative complications, after correcting for captured confounding factors. Although SES was not independently associated with OS, we observed a significant association between SES and CSS after correction for other known captured prognosticators (HR: 0.99 per percentile). This corresponds to a hazard ratio of 0.80 per quartile incremental SES increase. The current results should be viewed in the context of the Dutch equal access healthcare system. Therefore, the association between income and postoperative survival that was demonstrated in the present study cannot be attributed to inequality in healthcare resources. This is, to our knowledge, the first study to explore the influence of SES in a prospective cohort of consecutive colorectal cancer patients and stresses the importance of SES as a prognostic factor in these patients.

As in previous studies, our results indicate that SES is also associated with short-term outcome [17, 34]. This may partially be explained by confounding factors associated with both lower SES and higher postoperative morbidity. At baseline, diabetes mellitus was more prevalent in patients in the lower quartiles. This factor, as well as several others, such as liver disease, is incorporated in the ASA classification. These were significantly higher in the lower SES quartiles as well. Patients in the lower SES quartiles more often underwent open surgery, despite tumour characteristics being similar with regards to location, stage and pathologic prognostic factors. Moreover, patients were significantly older in the lower SES quartiles, and therefore a larger part of these patients may have been retired the year prior to surgery and have less income.

These differences in treatment, without apparent clinical explanation, mirror those described in previous studies.[34–36] Even after correction for known confounding risk factors including comorbidities, SES was a significant predictor for postoperative morbidity. These results suggest that the correlation between SES and postoperative outcomes is determinant upon factors that are currently not adequately considered or understood.

In general, long-term results presented in our study are in line with previous studies, showing impaired survival in patients with low SES [20, 22, 23, 37]. Several previous studies have offered explanations for this survival discrepancy in a setting of equal healthcare access [38–51]. These possible explanations include psychological factors, medication compliance and diet, exercise, air pollution, and even epigenetic factors [38–51]. Interestingly, in our study, the correlation between SES and CSS was more outspoken than the correlation of SES with OS (i.e. all-cause mortality), making some of these explanations less plausible. In addition, this suggests that the survival discrepancy between SES quartiles was not solely attributable to the unequal distribution of age and comorbidities between the groups, as these factors have a less obvious effect on cancer-related outcome. Fowler et al. showed that tumour stage and treatment contributed for a great part towards the difference in 3-month mortality between the most and least deprived patient groups [52]. Known and relevant tumour characteristics, as earlier described, did not significantly differ across SES quartiles. In contrast however, treatment strategies were different between groups. In addition to a different surgical approach between the quartiles, fewer patients of lower SES quartiles received neoadjuvant and adjuvant chemotherapy. The inequality of treatment with neoadjuvant therapy can partially be explained by differences in proportion of patients with rectal cancer between the SES quartiles. Neoadjuvant chemotherapy is only registered in patients with rectal cancer whereas adjuvant chemotherapy is only offered to patients with colon cancer. However, as in our study, previous studies show that lower-middle SES and low-SES patients were less likely to receive chemotherapy in general [53]. Furthermore, since SES remained significantly associated with CSS after correction for baseline variables, it is possible that some of the driving factors of treatment differences cause additional differences in strategy not captured in our cohort. Besides differences in the administration of chemotherapy, compliance with treatment may also play a role. Prospective studies will be required to elucidate the exact causal mechanism of this remaining correlation.

GHI was chosen as a surrogate marker for SES in this study, as it is a proven accurate reflection of SES-related health disparities [26, 29–31]. It was not adjusted to household size, as previous studies showed this adjustment did not improve predictability of the associated health disparities [30]. Other reported determinants of SES include the highest attained level of education, current occupation, parent’s education and occupation, and household conditions [34, 36, 54–56]. Unfortunately, no previous studies have compared the accuracy of these markers in a cohort of (colorectal) cancer patients, which limits their comparability [11–23]. Since GHI is a valid and readily available metric for all Dutch citizens registered by an independent organization (Dutch Statistics; CBS), we believe this marker adequately captures SES for our purposes. Use of this marker in different populations or different countries would add to the comparability of the plethora of SES studies currently being conducted.

Some inherent shortcomings of this study should be noted. First, with regards to patient factors, we were unable to determine several lifestyle factors of potential importance, due to the inherent limitations in our database. These include smoking, diet, and compliance with medication [44, 45, 57]. Even though these factors reportedly do not explain all differences, we believe correcting for them would have enhanced our results [30, 58, 59]. An additional drawback in our study was our inability to comment on exact healthcare consumption for CRC and comorbidities, limiting our ability of testing the premise of equal healthcare accessibility. Variation in SES between the hospitals can have some effect on the outcome; however, guideline adherence was equal across the hospitals plus complications and survival outcomes did not differ between the hospitals.

Finally, ethnicity could be a confounding factor with SES, and the unfavourable surgical outcome of GHI in this study might also partially be explained by differences in ethnicity. However, ethnicity is not reported in the Netherlands, which limits this study because no analyses can be performed on the actual relevance of this risk factor [37].

In conclusion, our results show that low SES is associated with worse outcome in patients undergoing curative surgery for stage I–III colorectal cancer. Future studies are required to elucidate the association between SES and survival in cancer patients, which suggest that SES encompasses risk factors and behaviours currently not adequately considered. Such studies would require additional power and potential explaining factors and would ideally be able to distinguish between the effects of patient and treatment related measures.
